# Snip conjunctivoplasty associated with methylene blue staining for treatment of postoperative conjunctival chemosis

**DOI:** 10.1080/23320885.2024.2306984

**Published:** 2024-01-25

**Authors:** G. Squarcia Neri, G. Pezone, A. Cavaliere, R. Lo Schiavo Elia, M. Sorotos, F. D’Andrea, F. Schonauer

**Affiliations:** a Unit of Plastic Surgery, University “Federico II,”Naples, Italy; b Ruesch Private Hospital, Naples, Italy; c Unit of Plastic Surgery, Faculty of Medicine and Psychology, Sapienza University of Rome - Sant’Andrea Hospital, Rome, Italy

**Keywords:** Blepharoplasty, lower lid blepharoplasty, upper lid blepharoplasty, conjunctival chemosis, blepharoplasty complication

## Abstract

Conjunctival chemosis usually undergoes spontaneous resolution; sometimes, it requires treatment. We present the case of a 43 years-old female patient who developed bilateral conjunctival chemosis following upper and lower blepharoplasty. Two months after the operation, patient underwent bilateral snip conjunctivoplasty with methylene blue demarcation of the chemotic conjunctiva.

## Introduction

The conjunctival chemosis is defined as the protrusion of the bulbar conjunctiva with a blister-like aspect due to the formation of an exudative collection; in most cases it is an acute and transient phenomenon but can become chronic and refractory to conservative medical treatments. Typical clinical manifestations include irritation, pain, foreign body sensation, epiphora and discomfort due to the cosmetic appearance [1]. The diagnosis is made by physical examination and with the aid of a slit lamp.

Severe conjunctival chemosis can present following eyelid surgery; the incidence following blepharoplasty varies from 1% to 12% [2–4] with higher frequency in lower blepharoplasty, especially when associated with fat removal [5]. When a canthopexy or canthoplasty is performed we can see an incidence of 11.5% to 12.1% [6].

Conjunctival chemosis can be classified in a four types system: Type 1, mild acute edema and inflammation with complete eyelid closure; Type 2, severe acute edema with inflammation that prohibits complete eyelid closure; Type 3, subchronic edema and inflammation that persists longer than three weeks; Type 4, chemosis associated with lower lid malposition [7].

The range of therapies available is wide; in addition to the more classic and conservative medical treatments, over time, various surgical techniques have been introduced.

We report a case of a conjunctival chemosis following upper and lower blepharoplasty successfully treated with a snip conjunctivoplasty associated with demarcation of the chemotic zone with methylene blue. This combined technique has allowed us to treat the pathology in an excellent and safe way, saving the normal conjunctiva by removing only the pathological one and ensuring a fast recovery.

## Case report

**Figure 1.**
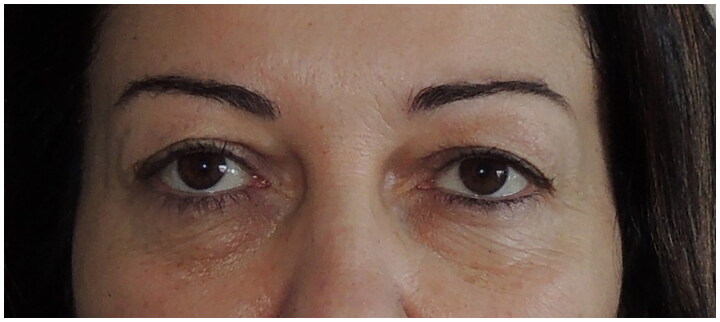
Preoperative aspect of the patient with dermatochalasis at upper eyelids and fat and skin excess at lower lids.

A 43-year female patient presented to the clinic with upper and lower dermatochalasis (Figure 1).

In February 2021 she underwent upper and lower lid blepharoplasty under general anesthesia. Upper lid blepharoplasty was performed after assessing the amount of excess upper eyelid tissue; after bilateral positioning of corneal protectors, skin excision was performed according to preoperative markings; lateral hooding was addressed and corrected with a lateral extension of the elliptical excision (Pastorek modification) [8]; a strip of orbicularis muscle was removed; after gentle blunt dissection, orbital medial fat pad protruding through the incision was removed using bipolar cautery. Resulting skin margins were approximated using a 6/0 Nylon running suture.

**Figure 2.**
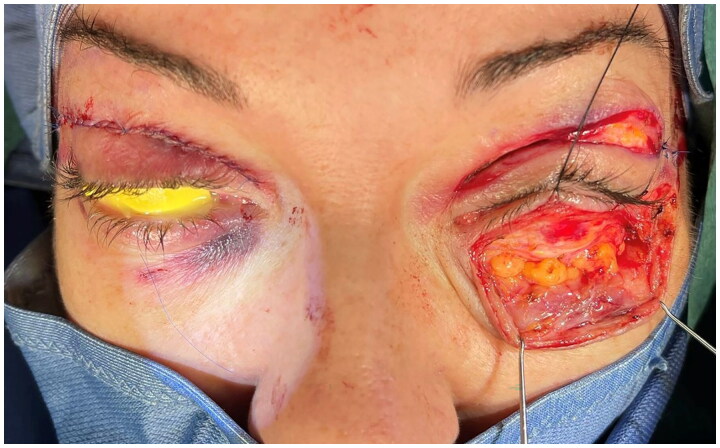
Intraoperative view of the fat compartments’ prominence of the left lower eyelid before conservative excision; corneal protectors were used during surgery.

Lower lid blepharoplasty was then performed with a transcutaneous approach: after subciliary approach, dissection was performed first in the subcutaneous plane, above the palpebral portion of orbicularis muscle, and then in the submuscular level, below the orbital portion of the orbicularis muscle. Dissection was carried out until the infraorbital rim was reached. Orbital medial fat pad was isolated (Figure 2) and removed conservatively with the use of bipolar forceps. Subsequently, excess skin was excised in a very conservative manner. A small lateral extension of the orbicularis oculi muscle was prepared to stabilize the lower lid before skin suture (Adamson’s flap) and then anchored to the periosteum of the lateral orbital rim using a 5/0 Vicryl. Skin was sutured using a 5/0 Prolene intradermal suture. 6/0 Nylon was used for the lateral wound interrupted stitches.

The day after the operation the patient was discharged home. Antibiotic ophthalmic ointment based on Chloramphenicol, Colistimethate Sodium and Tetracycline (Colbiocin; Sifi Spa) was prescribed for the first 72 h.

At the 72 h follow-up, the patient complained of irritation and foreign body sensation, while clinically a finding of bilateral conjunctival chemosis was noted. Tobramycin and Dexamethasone-based eye drops (Tobradex; Alcon Inc.) were then prescribed.

**Figure 3.**
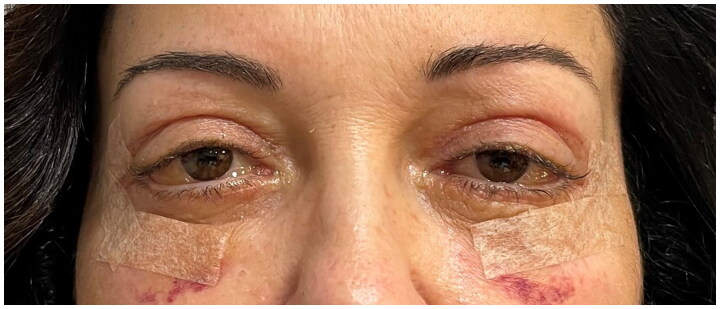
Bilateral conjunctival chemosis present at three weeks post operative follow-up.

Three weeks after surgery the conjunctival bilateral chemosis was still present despite medical therapy (Figure 3), with great concern of the patient; for this reason a more aggressive therapy based on isotonic ophthalmic solution eye drops with Hyaluronic acid sodium salt 30% and amino acids (BLUyal; Sooft Italia Spa), Betamethasone and Chloramphenicol eye drops (Betabioptal; Gekofar) was prescribed.

**Figure 4.**
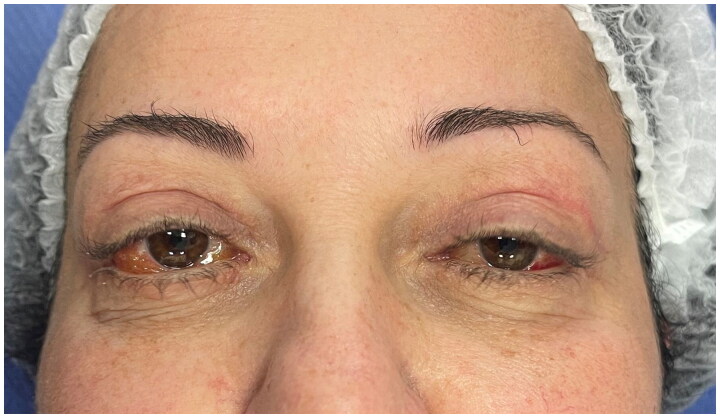
Chemosis was still present two months after upper and lower lid blepharoplasty.

**Figure 5.**
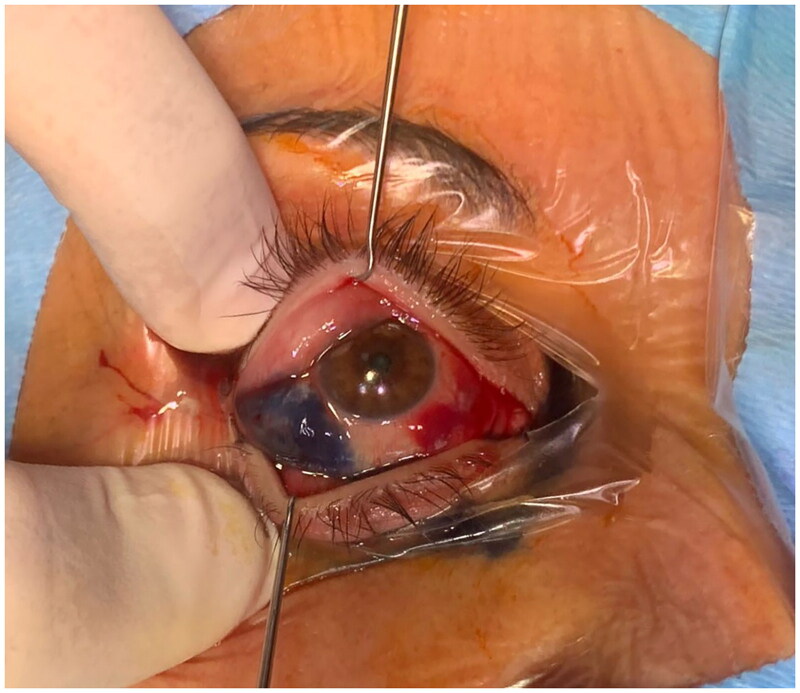
Intraoperative aspect of methylene blue demarcation of the chemotic area at right eye before snip conjunctivoplasty.

**Figure 6.**
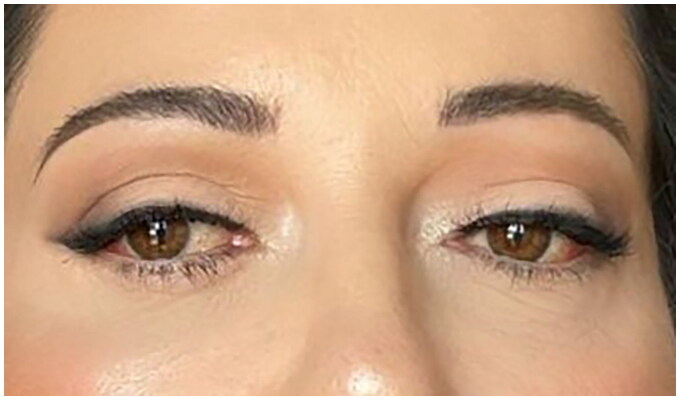
Six months follow-up showing stable resolution of the complication.

Two months after surgery, after failure of conservative therapy, persistence of the pathology was observed (Figure 4). At this stage a first surgical attempt was performed by an ophthalmologist consulted by our patient, who decided to perform a bilateral office needle aspiration of the serous conjunctival collections. After another 10 days, with no resolution of the complication, our team decided to refer the patient to our trusted ophthalmologist, who decided to perform a minimally invasive conjunctivoplasty. At surgery, under local anesthetic, after delimitation of the chemotic conjunctiva with Methylene blue, a minimally invasive conjunctivoplasty (snip conjunctivoplasty) [9] was performed by the ophtalmologist together with our plastic surgery team (Figure 5). Westcott scissors were used to excise a small strip of conjunctiva and Tenon capsule. Immediate release of subconjunctival fluid was noted; same procedure was performed at the left eye. Betamethasone and Chloramphenicol eye gel (Betabioptal; Gekofar) was prescubed for 7 days and then isotonic ophthalmic solution eye drops with Hyaluronic acid sodium salt 30% and amino acids (BLUyal; Sooft Italia Spa) for 21 days. Follow-up visits were scheduled at 3, 7 and 21 days postoperatively. At one-week follow-up, the chemosis had completely resolved. No recurrence was observed at six months follow-up (Figure 6).

## Discussion

Conjunctival chemosis represents one of the most frequent complications of blepharoplasty. Several factors have been associated with the risk of developing conjunctival chemosis: male gender, advanced age, hormone therapy, eyelid laxity, lagophthalmos, ocular allergy and dry eye syndrome are important predisposing factors that should be investigated in the patient’s preoperative history. At surgery, a crucial role is played by excessive cauterization, prolonged scleral exposure, manipulation of the conjunctiva, damage to the lymphatic and venous drainage system and the development of periorbital edema. Different prevention strategies can be adopted both in the operating room using corneal protectors, frequent moisturizing, judicious dissection, irrigation with cold saline solution and in the post-operative period such as wetting drops, ophthalmic lubricating ointment, ice packs, head elevation and compressive patches [4,7,10].

In most cases chemosis spontaneously resolves in two months without permanent sequelae [10]; topical lubricant, ocular decongestants, anti-inflammatory and pressure patching represent the standard therapeutic options in the first one to three postoperative weeks. Another method is patient finger compression of the eyelid over the chemotic area [11].

However, sometimes, chemosis does not spontaneously resolve and become chronic and not responsive to conservative management; at this point surgical treatment becomes necessary.

There is no gold standard treatment for persistent chemosis after eyelid surgery [12]; different surgical interventions have been introduced including lymphatic drainage, drainage conjunctivotomy, silicon bolster in the lower fornix with a tarsorrhaphy suture, plication of the redundant conjunctiva with 6-0 Vicyl [6,13], perilimbal needle manipulation [14], high-frequency radio wave electrosurgery [15], hand-held fine-tip cauterization [12], subconjunctival injection of sclerosing materials like Tetracycline 2% [16] and different methods of conjunctivoplasty.

Over time, various conjunctivoplasty techniques have been described; in 2005, Thakker et al. performed a limbal peritomy and subconjunctival and sub-Tenon’s fascia dissection with a 6/0 suture to close the peritomy [5]; in 2010, Jones et al. introduced a new less invasive technique called snip conjunctivoplasty characterized by the excision of a small elliptical strip of conjunctiva and Tenon’s capsule at the inferior aspect of the chemotic conjunctiva without placing any suture, allowing the fluid to drain [9].

As far as the side effects of the different techniques are concerned, we can see how in the techniques characterized by the use of sutures and foreign bodies such as silicone bolsters there is an increased risk of inflammation, infection and discomfort while, in the sutureless ones, beyond the risk of uncontrolled bleeding, there is the possibility of a residual subconjunctival dead space where extracellular fluids could accumulate again.

Snip conjunctivoplasty is a minimally invasive option because excising a small strip of conjunctiva and Tenon ‘s capsule slightly reduces the redundant conjunctival tissue, tightens its surface area and let the fluid drain without the use of sutures, thanks to the conjunctiva capacity to regenerate itself, healing by secondary intention, thus avoiding the risk of infection, inflammation and discomfort [17,18].

This minimally invasive approach has proven to reduce the risk of complications such as hypertrophic scarring, conjunctival fornix adhesions and fibrosis.

In our case, we decided to perform a snip conjunctivoplasty after staining the chemotic zone with methylene blue for detecting its precise extension. The use of vital dyes is a common practice in conjunctival surgery for locating the margins of a lesion as it facilitates its complete intra-operative removal and also aids in successful reconstruction of the remaining conjunctiva [19,20].

With this surgical approach, chemosis had successfully resolved without persistence of further symptoms; no recurrence was observed in subsequent follow-ups. The combination of two techniques allowed us the lowest surgical invasiveness and a rapid recovery, minimizing the risk of damaging the healthy conjunctiva.

## Conclusion

Snip conjunctivoplasty technique associated with the use of methylene blue represents an excellent surgical approach for the treatment of refractory postoperative conjunctival chemosis.

The simplicity of execution and its replicability together with the effectiveness, the low invasiveness and costs, represent the strengths of this combined surgical technique.
